# Chinese- and English-speaking adult current smokers’ perspectives on smoking and culturally and linguistically appropriate cessation: a qualitative analysis

**DOI:** 10.1186/s13722-020-00197-4

**Published:** 2020-07-06

**Authors:** Iraj Poureslami, Jessica Shum, Niloufar Aran, Noah Tregobov

**Affiliations:** grid.412541.7 0000 0001 0684 7796 UBC, Faculty of Medicine, Respiratory Medicine Division, Centre for Clinical Epidemiology and Evaluation (C2E2), Vancouver General Hospital – Research Pavilion, 716-828 West 10th Ave, Vancouver, BC V5Z 1M9 Canada

**Keywords:** Smoking perception, Smoking cessation, Chinese-Canadian population, Risk perception

## Abstract

**Background:**

A lack of culturally and linguistically appropriate smoking cessation intervention programs exist among Chinese-Canadian communities. Smoking cessation programs that are provided in Canadian mainstream culture and language have shown limited effectiveness in altering smoking behaviours of smokers from these communities. Our study aimed to explore and compare smoking patterns, knowledge, beliefs, and risk perceptions of adult current smokers between Chinese- and English-speaking Canadians participating in a culturally and linguistically tailored smoking cessation program.

**Methods and Design:**

A qualitative study embedded in an effectiveness study using an 8-month quasi-experimental design, was conducted to compare the effects of four one-on-one culturally and linguistically sensitive consultation sessions (intervention group) and three telephone follow-up assessments (control group). All participants were provided take-home educational materials (designed exclusively for this study), and completed study questionnaires at baseline and 6-month post-intervention. An 8-month post-intervention phone assessment was conducted with all participants to assess cessation progress and maintenance.

**Participants:**

70 Chinese- and English-speaking adult (aged 19-80) current smokers (≥ 5 cigarettes per day) residing in the Greater Vancouver Area, Canada, were recruited between May 2018 and April 2019.

**Data analysis:**

Thematic analysis was conducted on self-reported qualitative information from study questionnaires and verbatim transcripts of in-person consultations and telephone follow-ups. Cultural- and demographic-related themes were considered.

**Results:**

Perceptions of smoking patterns, smoking status, triggers, and barriers to smoking cessation were identified. Important elements of smoking cessation program, including facilitator characteristics, duration, procedures, cultural factors, and topics were also identified. Differences in perceptions of smoking were observed between gender and language groups. Stress was a major trigger for smoking in both language groups. An individual’s social network was reported as the largest barrier to successful cessation for Chinese-speaking participants.

**Conclusions:**

Our study provides knowledge and information to further examine the role of risk perception (realization of the possible harms of smoking) in smoking cessation to facilitate the development of future interventions that could more effectively promote smoking cessation among new immigrants and within ethnocultural communities. We found that our program was generally accepted by smokers in both language groups and the participants reported that they were able to apply the strategies learned in the intervention during their quit smoking plan.

## Background

Smoking continues to be the leading cause of preventable deaths worldwide, accounting for over 7 million deaths each year [1]. Tobacco use accounts for more than 45,000 deaths in Canada annually [2, 3]. The 2012 Health Canada data show that over 18% of deaths in Canada are attributable to smoking and exposure to second-hand smoke [3]. Although smoking rates have been reported to be increasing among individuals in low- and middle-income countries [4], in recent years, the prevalence of smoking among Canadian immigrant groups from these countries is generally unknown [5]. The perceived risks and harms of smoking as well as potential barriers or facilitators to smoking cessation within the Chinese-Canadian population—one of the fastest growing ethnic groups in Canada [6]—is understudied [7]. When considering recent (within the previous 5 years) Chinese immigrants to Canada, there are both subjective cultural and gender characteristics related to self-reliance and self-control that may influence one’s attitude and willingness to participate in smoking cessation programs or seek professional assistance for their cessation, as smoking is a gender-related issue in these communities [7]. Studies by Mao et al on current Chinese-Canadian smokers indicated that the most prominent barriers to smoking cessation are the lack of available cessation programs and information as well as the difficulty accessing smoking cessation assistance [7–9]. Studies conducted in China and the USA have indicated that a lack of English language proficiency, low education attainment, and cultural norms and practices, are associated with higher levels of smoking among Chinese-American men, in comparison to native English speakers [8–11]. These factors pose additional barriers to smoking cessation since limited English proficiency is a noted factor towards reduced access to smoking cessation services within an individual’s community [10, 11]. Furthermore, a cultural norm that promotes male smoking patterns and diminishes female smokers may also lead to social pressures that prevent enrollment and active participation in a smoking cessation programs, as reported by studies conducted in Mainland China and Hong Kong [12–14]. As smoking by females is generally not accepted within Chinese cultural norms, the majority of Chinese-speaking female smokers are hidden or act as ‘hidden smokers’ [15, 16]. These Chinese-speaking female smokers are also less likely to seek assistance for cessation when compared with their male counterparts [17]. These underlying cultural attitudes may contribute to the understudying of women from this group. Other studies [18, 19], including our previous research [20–22], have found that Chinese immigrants to Canada are less likely to receive and consider professional smoking cessation advice from a physician in their new country, as well as information regarding the risks of smoking continuation when compared to their native English-speaking counterparts. This cycle is perpetuated when Chinese immigrants do not consider the physician’s advice and the information lacks cultural or social relevance [21, 22]. This barrier may negatively influence their understanding and knowledge of the link between smoking and chronic diseases.

Current smoking cessation programs used in clinical practice typically involve self-help strategies, pharmacotherapy, and less frequently, behavioural counselling [23, 24]. Analyses of these programs have concluded that personalized approaches, namely individualized counselling-based therapies, have been highly effective in assisting cessation and helping to empower smokers with the necessary tools to combat the challenges of quitting [24–26]. There is a lack of culturally and linguistically appropriate programs that promote smoking cessation in Chinese-Canadian communities and a gap in the understanding of the differences between this group and English- or French-speaking Canadians [20–22]. Accessibility to smoking cessation programs that are provided in Canadian mainstream culture and language (English and French) have shown limited effectiveness in altering smoking behaviours of smokers from ethnocultural communities [6, 8, 9, 27].

It is evident that there is a need to develop and implement community-driven, culturally competent smoking cessation programs for ethnocultural communities; especially communities with members who exhibit relatively high prevalence of smoking behaviours—namely Chinese immigrant communities [20–22]. These programs should be developed with enhanced consideration for the impact of social influence, the difficulties associated with adjusting to a new social environment, and they must also address the culturally-rooted stigmatization of smoking [9, 21], primarily among female smokers. These social barriers could be addressed at the community level through targeted interventions that seek to understand individual smokers and thus alter prevailing negative cultural attitudes and culturally-based stigmas.

Our team developed and tested the effectiveness of a newly developed, culturally and linguistically comprehensive smoking cessation program among Chinese-speaking (Mandarin and Cantonese) and native English-speaking Canadians in the Greater Vancouver Area, Canada, using a community-based pilot intervention study. The primary aim of our study was to help participants cut down or quit smoking over an 8-month study period by applying a combination of pharmacotherapy (e.g., Nicotine Replacement Therapy (NRT)), educational intervention, and behavioural counselling, with an enhanced focus on perception modification using a risk perception (realization of the possible harms of smoking) approach. Our objectives were: (1) to develop a professional, culturally and linguistically based smoking cessation program; (2) to better understand and compare the tobacco use, knowledge, beliefs, and perceptions of current smokers from the target communities; (3) to test the effectiveness of participating in a culturally and linguistically relevant smoking cessation counselling program coupled with access to comprehensive educational materials; and (4) to empower smokers to make sound decisions about their smoking habits and cessation (improved self-confidence) via the proposed intervention. To achieve the goals of this feasibility study, we incorporated social and cultural factors that were critical for Chinese-speaking smokers, as suggested by our current and past study participants, throughout our study (e.g., during the development of educational materials, consultation sessions, assessments, etc.). These cultural factors offered consideration for Chinese values, norms, and challenges, such as: the social acceptability of smoking, coping with immigration-related stress, a limited availability of social support, challenges with language proficiency (e.g., communicating in a secondary language), and stigma associated with accessing smoking cessation services (a potential barrier to smoking cessation). In this paper, we report on the qualitative results of our study.

## Methods

### Study design and procedure

**Fig. 1:**
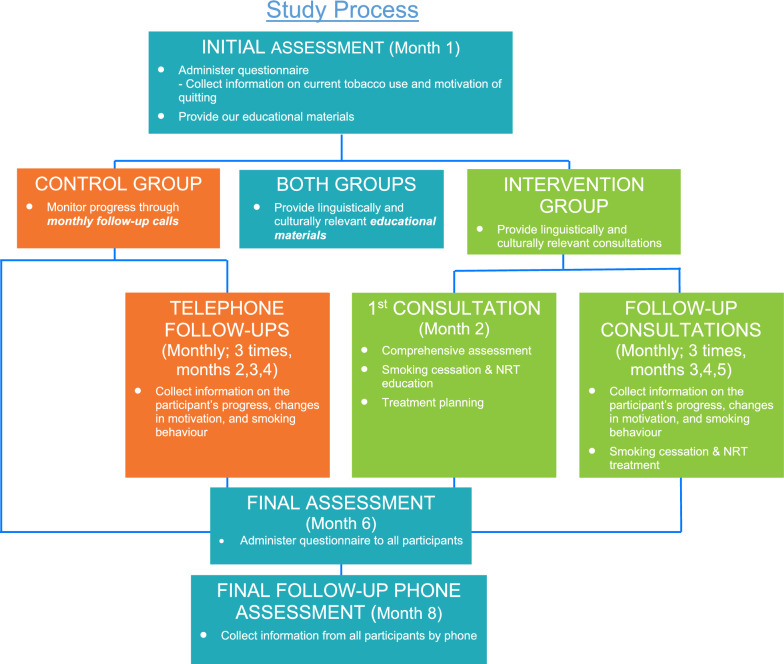
Study process

A quasi-experimental study design, with a baseline assessment and several follow-up measures, was applied. As shown in Fig. 1, eligible participants were given the choice to either participate in the intervention group (4 individualized smoking cessation consultations and an educational package) or control group (received educational package alone). Random assignment was not feasible for our study due to time commitments and obligations of participants. Therefore, we left assignment of study group to the choice of the participants. For both study groups, the 8-month study period included two in-person assessments (baseline and 6-month post baseline assessment) and one telephone assessment at 8-months post baseline. A standardized written study questionnaire was administered to participants in both groups at the baseline and 6-month assessments. Participants completed the questionnaire independently and in their preferred written language. The baseline and 6-month assessments captured potential changes in smoking patterns, risk perceptions, and smoking behaviours. In addition to the two in-person assessments, the intervention group received 4 in-person consultation sessions, which occurred over a period of 4 months (at months 2, 3, 4, and 5), while the control group received three telephone follow-ups at months 2, 3, and 4 to track their cessation progress, without provision of consultation/advice or intervention. During both the in-person consultations (intervention group) and the telephone-follow-ups (control group), the counselor or research assistant administered a checklist of questions to both groups (either in-person or over the phone) regarding cessation progress and perceptions that participants responded to verbally and in their preferred language. A bilingual member of the research team translated participants’ responses on the checklists. The consultation sessions and telephone follow-ups started one-month post baseline assessment. Finally, an 8-month follow-up call was conducted with all participants to assess self-reported progress towards their individualized goal of cessation or reduction, their self-confidence to maintain the cessation or reduction goal, and any suggestions they may have to improve our smoking cessation program.

### Study sample, eligibility and recruitment

English- and Chinese- (Mandarin and Cantonese) speaking current adult smokers (≥ 19 years of age) who smoked five or more cigarettes a day were recruited and enrolled from clinical settings as well as through community organizations serving immigrants to the Greater Vancouver Area. In addition, relevant social media platforms (e.g., Vanpeople, Vansky) were used to recruit smokers from the target communities. Participants recruited from clinics did not receive any advice from their physicians to participate in the study. Furthermore, we posted our recruitment flyers on the British Columbia QuitNow program’s Facebook page. Participants also had to be willing to quit or reduce their smoking behaviour, as well as participate in a smoking cessation program for the duration of 8 months. All participants reviewed and signed an informed consent form provided in their preferred language (Chinese Simplified, Chinese Traditional, or English) and were encouraged to ask any questions before agreeing to participate in the study. Prior to the study, we hypothesised that fewer females would participate in the study due to the nature of smoking practices in the Chinese community as female smokers are often stigmatized [15, 16] and face social barriers to accessing professional cessation assistance [17]. However, the number of female participants in our study (n = 20) provided sufficiently rich data to explore differences in smoking perceptions and behaviours between genders.

### Development of study materials

Our study applied a community-based participatory research (CBPR) approach in the development of the study materials and intervention. A CBPR approach was used to ensure that community members and key-stakeholders were involved in the development and implementation of the study, and that their insights into and knowledge of the target community were considered when developing study protocols and materials. Smoking cessation educational materials (e.g., educational videos and readings) were developed in English and then translated to Chinese Simplified and Traditional formats during the pre-intervention/development stage of the study (2014–2016) with engagement and insights from smokers of the target communities [20–22]. In addition, a number of consultation materials (e.g., individualized quit smoking action plans and cessation-related materials) and study documents (e.g., recruitment flyers and pamphlets) were developed for participants and made available in all three languages. The educational materials were tailored to address the target communities’ prevailing beliefs and practices regarding smoking and smoking cessation. Throughout the development stage, there was enhanced consideration for the target communities’ norms and perspectives to ensure cultural and linguistic appropriateness.

### Intervention group procedures

The intervention consisted of four 60 min in-person counselling sessions with an experienced bilingual (English-, Mandarin-, or Cantonese-speaking) smoking cessation counsellor. In total, there were four smoking cessation counsellors on the study team: a registered nurse, a medical doctor trained in addiction services, a counsellor with a background in psychology, and a foreign medical doctor from China. The registered nurse and medical doctor trained in addiction services were English-speaking employees of an existing Smoking Cessation program in the Greater Vancouver Area. They were recruited for this study and trained the counsellor and foreign medical doctor on how to provide consultations. The counsellor and the foreign medical doctor were Chinese, and both spoke Cantonese and Mandarin fluently. During each session, the consequences of smoking and the effects of tobacco on health, the addictive nature of nicotine, the socio-environmental factors affecting smoking and smoking cessation, smoking pattern and triggers, how to overcome withdrawal symptoms, and participants’ own experiences with smoking were discussed. During the consultation, a standard checklist was used by the counsellor to collect participant’s verbal responses to the discussed topics. The information was then translated to English on the same checklist by the bilingual counsellor who collected the information. The aim of the intervention was to empower current smokers to make sound decisions about their smoking behaviours and cessation (self-efficacy), as well as improve self-confidence to quit. The consultation sessions were tailored to each participant’s specific needs and smoking habits. The participants were encouraged to invite a close friend or family member to the session to increase proximal social support. The educational materials provided to participants included audio-visual DVDs and pamphlets addressing issues related to smoking behavioural modification as well as two smoking cessation booklets in the participant’s preferred language (Mandarin, Cantonese, or English).

### Control group procedures

The control group participants attended one in-person orientation meeting to learn how to apply the self-help educational materials at home and received three 10–15 min follow-up calls (approx. once each month) to monitor their progress. During the phone follow-up assessments, any changes in participants’ quit plan, triggers, smoking pattern, self-efficacy to quit smoking, and current smoking status were recorded on a checklist by a bilingual research assistant following a standard script. The research staff conducting the call also answered participants’ questions about their quit journey, NRT, and withdrawal symptoms. Modest incentives were paid to both groups to compensate for time, travel, and parking expenses for each in-person visit.

### Data collection and study measurement tool

We elicited participants’ perspectives using four qualitative data sources: (1) a study measurement tool in the form of a questionnaire that was administered to the intervention and control groups at the initial and 6-month assessments; (2) verbatim quotes from telephone follow-up assessments with the control group participants; (3) verbatim quotes from in-person consultation sessions with the intervention group participants; and (4) verbatim quotes from final follow-up phone assessment (8-month) with all participants. The measurement tool used for this study included 96 items (both qualitative and quantitative). An initial version of the tool had been developed and tested in our previous study [21], see Appendix A. Additional items and necessary modifications were applied to include demographics and smoking history, smoking pattern, smoking triggers, stages of change, history of using smoking-related pharmacotherapy, and current smoking status. In addition, in the absence of well-established risk perception scales for smoking, measures specified to risk perception and self-efficacy were added into the measurement tool to assess participants’ overall perceptions of smoking (e.g., positive vs negative, addiction vs habit, etc.) as well as benefits vs. harms of quitting or continuing smoking. The self-efficacy items included two questions on the importance of smoking cessation and confidence in quitting smoking on a scale of 1 to 10 (*e.g., How important is it for you to maintain cutting down or smoke free?* (*Please indicate your score on a scale of 1*–*10, with 10 being the most important/confident*)). Examples of the risk perceptions questions included: *What is your perception of smoking? (i.e., What do you think smoking is? What do you know about it)? (Please write it in your own words); What effect(s) do you think smoking has on your health?; What do you think are the disadvantage(s) to you of smoking cigarettes?; In your opinion, do you think there are any benefit(s) to the society if smokers attempt to quit? (Please write it in your own words); In your opinion, do you think there are any harm(s) to the society if smokers continue to smoke? (Please write it in your own words).* The 8-month telephone follow up assessment consisted of questions on: changes in smoking perception and smoking status since the last in-person assessment (6 months). Participants’ self-efficacy was measured by asking two questions about their perceived importance and confidence to quit smoking (*i.e., how confident are you in maintaining cut down or smoke free? how important it is for you to maintain cut down or smoke free?*). We also asked their feedback on the study.

### Qualitative data analysis

Bilingual research assistants translated study questionnaires responses provided in Chinese Simplified or Traditional to English. Likewise, the participants’ responses to the question checklist from consultations or telephone follow-up assessments were transcribed and translated (when necessary) to English by the same bilingual research assistants. The data were then imported (in English) into the Navigating Viewpoints, Images and Value Observed (NVivo) software version 12, a qualitative data analysis software [28] by an experienced member of the research team (JS). All qualitative data from the aforementioned sources were included in the analysis. Data were coded, and thematic analysis was conducted to categorize and develop themes via constant comparison across nodes. We applied a constant comparison approach to systematically organize, compare, and understand the similarities and differences between perceptions of the participants. An inductive approach was applied for themes. Descriptive saturation was reached when no new codes or themes emerged from further analysis. One of the authors of the manuscript (JS) coded the data, which was then reviewed and checked by the principal investigator (IP). IP also provided input on the coding framework and any disagreements or discrepancies were discussed between the coders and the rest of the research team. Any necessary modifications to the coding framework were then applied. JS and IP met on multiple occasions to discuss the emerging themes that resulted from the analysis and their relationships.

Participants’ progress to smoking cessation was captured by the difference between their self-reported smoking at baseline, 6-month, and 8-month assessments. Their smoking status was then classified under one of three categories: no change; smoking more; and reduction in the amount or quit smoking.

## Results

Between May 2018 and April 2019, 78 current adult smokers were recruited and enrolled in the feasibility study, from which 70 participants completed all in-person and telephone follow-up assessments. 8 participants were excluded from participating in the study (4 because of transit and parking issues, 3 declined to participate after enrolling, and 1 moved out of the area and was unable to attend the sessions). Table 1 summarizes the recruitment sources for the 70 participants. Table 2 describes characteristics of the study sample for Chinese- (43%, n = 30) and English-speaking (57%, n = 40) participants. The participants were predominately male (71.4%, n = 50). Participants were asked demographic information such as age, gender, education level, language spoken at home and in the community, cultural affiliation, and occupation status at baseline, as we were interested in studying both socio-demographics as well as the cultural aspects of smoking.

**Table 1:** Sources of participants

From clinic (30%)	Lung clinic	21	30%
From community (70%)	Social media (Craigslist/Vanpeople/Vansky)	21	30%
	Referred by participant	9	12.5%
	Referred by staff	8	11%
	VCH (e-blast)	4	6%
	Promotional flyer	4	6%
	Community/Professional organizations (e.g., REACH, MOSAIC)	2	3%
	Other (e.g., past studies’ participants)	1	1.5%
	Total	70	100%

**Table 2:** Participant characteristics (N = 70)

Characteristic	Control group N = 29 (41.5%)	Intervention group N = 41 (58.5%)
Language	English N = T15	English N=25
	Chinese N = 14	Chinese N = 16
Gender	Female N = 6	Female N = 14
	Male N = 23	Male N = 27
Age	19–35 years N = 16	19–35 years N = 15
	36–55 years N = 9	36–55 years N = 19
	> 55 years N = 4	> 55 years N = 7
Education level	High school graduate or less N = 9	High school graduate or less N = 15
	Diploma after high school N = 8	Diploma after high school N = 12
	University degree or higher N = 12	University degree or higher N = 14

The qualitative data generated by the NVivo analysis is summarised below:

### Theme 1: Perceptions of smoking

The participants were asked to explain their thoughts and perceptions regarding smoking. The responses were mixed; however, most individuals in both the Chinese- and English-speaking groups described smoking as something negative (e.g., as a bad habit, an addiction, harmful to one’s health, etc.) with some mentioning the positive aspects of their behaviour. General perceptions and thoughts on smoking were categorized into 4 subthemes: (a) feeling stigma or shame; (b) tobacco cigarettes vs. others (e.g., cigars, e-cigarettes, marijuana); (c) denial of addiction; and (d) perceived positive effects of smoking. Table 3 illustrates participant quotes related to the 4 subthemes.

**Table 3:** Additional quotes from participants

*Theme 1: Perceptions of smoking*
Feeling stigma or shame “I feel trapped by them [cigarettes] and that makes me feel out of control”.—*Female English-speaking*
*Theme 2: Changes in smoking status among participants*
“And the closer I get to quitting, the more I enjoy every cigarette. It’s been my constant companion since I was 15 and now I’m in 73 now. So—I just love it”.—*Male English-speaking* “The positive is that it feels good when I have a cigarette. I feel relaxed. The negative is that I have asthma and I know sooner or later it’s going to catch up to me and my lungs. I feel it. Cause I quit smoking. I have quit and started. When I quit I feel really good and then I started I still felt good but now during the change in season, I’m starting to feel my lungs are little more congested”.—*Female English-speaking* “Bringing it to the brink of quit—that’s the hardest part. Because you’ve changed how you think about things when you go from 30 to 1 (referring to cigarettes). There’s a whole process of bringing it down to get ready. that’s the brink”.—*Female English-speaking*
*Theme 3: Triggers*
Bad mood “I don’t smoke at work and it makes me moody. I try to do something else”.—*Male Chinese-speaking* “I mean—I had gone through smoking cessation groups and this program and that program, and this thing and that things; like I tried everything. And I was done. But I couldn’t imagine life as a non-smoker”.—*Female English-speaking* In combination with drink or meal “I enjoy it with a glass of wine”.—*Female Chinese-speaking*
*Theme 4: Barriers to cessation*
Lack of stress management skills “When I get stressed out and anxious and when I get anxious I find that it (smoking) calms me down. I used sort of to self-medicate. And it just helps that way. I’ve tried many different patches. Like I said, I tried that for a year and then when I start drinking, I end up with a pack of cigarettes and a way I go again”.—*Male Chinese-speaking* “I quit a number of times, then something stressed me out and I started smoking. But every time, after the cessation group I did, I thought about every cigarette I think about what I am doing? I like smoking but everything but everything about it is bad… in my head”.—*Female English-speaking* “I want to quit, but it was a physical addiction you crave it and you get quite stressed if you couldn’t have a smoke”.—*Male Chinese-speaking* Lack of motivation to quit “It’s something I cannot let go off and it’s on my mind even when I quit sometimes, it’s a steady awareness when I’m around smokers you know and I think a big part why it’s an addiction is no matter how healthy I get I still want to go back to it sometimes and I can rationalize a reason to smoke”.—*Male English-speaking*

#### Feeling stigma or shame

The participants expressed feelings of shame for smoking, often stemming from the current cultural stigmatization of smoking. They mentioned that they did not like to smoke in front of other people because they found it embarrassing and an unhealthy habit: “*I have to leave my social gatherings to go smoke, I don’t like to smoke in front of others*”, “*I worry about kids in my neighborhood seeing me smoking outside*”. One factor contributing to the feelings of guilt and shame included the participant’s profession. One individual working in a health care setting said that her occupation had caused her to be a ‘secret smoker’. The stigmatization of smokers and being afraid to smoke in public were mentioned by female participants more than male participants. One English-speaking female participant mentioned: “*I know other people notice the smell of cigarettes on me*”. Other instances of feeling guilty or embarrassed were when participants were unsuccessful at their attempts to quit or reduce smoking during the course of the study, which caused them to feel like they had failed. A female English-speaking smoker said: “*It is very bad for health; it’s an addiction*—*losers smoke*”.

#### Tobacco cigarettes vs. others (e.g., cigars, e-cigarettes, marijuana)

A number of participants compared the smoking of traditional tobacco cigarettes to other substances such as cigars or marijuana. The majority believed that smoking non-traditional alternatives to cigarettes were less harmful than smoking traditional cigarettes: “*[Smoking] cigars are less harmful since it is not being inhaled into the lungs*”. Cigars were considered a healthier alternative to cigarettes as they were thought to cause less phlegm. One English-speaking female participant mentioned that she felt ‘smoke-free’ when using e-cigarettes as she believed some e-cigarettes do not contain nicotine at all. This was similarly mentioned by another Chinese-speaking male participant. Finally, tobacco cigarettes were also compared to marijuana; two male participants (one Chinese-speaking and one English-speaking) felt that marijuana was less harmful and addictive than tobacco cigarettes due to lower carbon monoxide levels and its perceived cleansing effects: “*It [marijuana] clears out my lungs*”.

#### Denial of addiction

Many participants in both language groups believed smoking was an addiction; however, there were misinterpretations of the harms of smoking, almost exclusively among heavy smokers (those who self-reported smoking more than a pack a day). Many participants believed they could control the addiction aspect of smoking, as they perceived it to be a habit. A male Chinese-speaking smoker said, “*Smoking is just a habit. Regular smokers’ blood [is] composed with nicotine and is forming a dependency*”. Another male Chinese-speaking participant tried to justify his habit, while mentioning the harms of addiction: “*I think smoking is OK as long as it doesn’t make me dependent to nicotine*”. Also, a female English-speaking smoker mentioned: “*To me, when smoking becomes a habit or routine, is a bad habit*”. A male English-speaking smoker said, “*Long*-*time* s*moking is harmful when you’re addicted to nicotine and cause health problems*”. Other participants believed that the body ‘needed’ nicotine. This was primarily mentioned by those who had smoked for many years and felt that cigarettes were now ‘a part of their life’. To quote one English-speaking male participant, “*My friend told me to never quit smoking, because it will kill me*”. This perception was noticeably more consistent with English-speaking female participants, and overall, English-speaking smokers believed their body needed nicotine more than Chinese-speaking smokers. Moreover, a few of the participants referred to their smoking as an addiction forming dependency.

#### Positive effects of smoking

Many participants also mentioned perceived positive aspects of smoking as a stress reliever (mental relief and calming agent) that helped them to relax. A male Chinese-speaking smoker said: *“Smoking for me is to alleviate stress, something that is an outlet for me to let go. For example, when I’m happy, excited, et cetera”.* Also, a Chinese-speaking female mentioned: *“It’s lighter. It stimulates the dopamine secretion which makes me feel happy”.* An English-speaking female stated that: *“I know smoking is harmful to my health and to other people, [but] smoke a cigarette is my first choice when I’m not feeling good. It will ease [sic] my emotion quickly”.* Interesting to note, female participants believed that smoking relaxed them to a greater extent (i.e. more forcefully) than male participants, who believed smoking helped them to release stress, improve concentration, and have an enjoyable time with friends.

### Theme 2: Changes in smoking status among participants

Participants’ smoking status was classified under three major categories: (a) no change; (b) smoking more; (c) reduction in the amount or quit.

#### No change

Three participants reported still smoking the same number of cigarettes as they did before. For those who had self-reported no progress towards quitting or reduction, the main reason was that they did not attempt any of the strategies discussed and suggested by the counsellor during the individualized consultations (e.g., nicotine replacement therapy or strategies to deal with triggers): “*I would really like to end this habit but do not seem to be able to get there*”. However, despite the lack of progress, these participants noted that they had become more conscious of their smoking habits and felt more informed to attempt quitting or reducing smoking at a later date.

#### Smoking more

Three smokers indicated they had increased their cigarette intake. The main trigger for the increase in smoking was stress: “*I’m smoking much more now, I’m dealing with a lot of stuff*”. Other reasons for smoking more included social events with friends, unemployment, and in those who had a habit of smoking while consuming alcohol.

#### Reduction in the amount or quit

Sixty-four participants in our study self-reported that they had either reduced their smoking intake (decreased to at least 1 cigarette/day less) or quit completely by the final in-person consultation. The approaches that had been applied to reduce the number of cigarettes included NRT, practicing positive self-talk, undergoing physical activity, changing their physical environment, buying cigarettes less often, and slowly cutting cigarettes out of their daily routine. These approaches were also mentioned as useful ways to deal with triggers or urges. Withdrawal symptoms were mentioned by those who had reduced their cigarette intake or quit smoking: “*I have a really increased appetite these days. I felt so hungry at 12 am after [I] had 2 meals around 6* *pm today*”. In addition, increased appetite and weight gain, feeling irritated and frustrated, difficulty sleeping, mild headaches, and having low energy were cited as withdrawal symptoms and effects. Only one Chinese-speaking male participant mentioned a financial improvement (realizing they had more savings and change on hand compared to before) due to reduced cigarette consumption. Health improvements after reducing or quitting smoking were reported in both language groups. The participants noticed physical and mental changes to their bodies such as becoming more physically fit, better breathing, less phlegm and dry throat, better concentration, and having more energy.

### Theme 3: Triggers

Multiple triggers were identified from the analysis including: (a) bad mood; (b) breaks at work; (c) friends smoke/social activities; (d) in combination with a drink or meal; (e) stress; and (f) time of day. The top three triggers for both English- and Chinese-speaking females were stress, the need to concentrate, and boredom. For males in both language groups, they were stress, having a smoke with friends, and needing to calm down.

#### Bad mood

Being in a bad mood or bored was one of the most common triggers to smoke for the participants. Some participants associated being bored with different moods such as feeling ‘lazy’, ‘lonely’, ‘depressed’, or ‘empty’. For example, “*I know I smoke because I’m bored and lonely sometimes*”.

#### Breaks at work

Smoking was often cited as a tool to take a break from work or an activity. The situations for smoke breaks varied from smoking with friends, co-workers, filling in gaps during down hours, to ‘just needing a break’. Multiple Chinese-speaking male participants mentioned that smoking during breaks helped them to concentrate and empty their mind of overwhelming thoughts: *“[Smoking is] a break, a moment to disengage from reality and re*-*attend to task, or rest*”. Smoking was used in the workplace to project a particular image of oneself to others: “*[Smoking] makes people look more mature [more responsible and reliable], help[s] people clear their mind*”.

#### Friends smoke/social activities

Having friends who smoked or took part in smoking, as a social activity or gathering was a major trigger for smoking in both language groups: “*Smoking is as a mean [sic] to socialize with others in my community*”. A few of the participants mentioned that they would generally not smoke if they were alone. Likewise, peer pressure or friend offering cigarettes was another reason for smoking initiation. Most participants in both language groups felt unable to refuse a cigarette when they were around people who smoked or when they were offered one by these people. The participants in our study mentioned experiencing cravings when they were around friends or co-workers who smoked.

#### In combination with drink or meal

Having a morning coffee was a trigger for smoking and was often described as part of the morning routine. A Chinese-speaking male participant mentioned that his morning smoke usually came with his morning coffee, and that this coffee was the most important part of his morning routine. Another Chinese-speaking male participant described the morning cigarette and morning coffee as a “*little ceremony*” to him. Alcohol often triggered individuals to reach for a cigarette: “*I’m drinking so the smoking goes up*”. With regards to eating, participants reported that they frequently smoked after a meal and rarely before the meal.

#### Stress

Stress was the number one trigger for many participants in our study to smoke. The stress that participants associated with smoking ranged from work or school stress to stress from health issues (themselves or others) to relationship stress (family or friends). This was mentioned equally by members of both the English- and Chinese-speaking communities. Immigration stress (e.g., language, cultural barriers) was another major influence mentioned by participants: “*[Smoking is] to take a break from life’s stresses*”, “*Smoking is something you do to help deal with the burdens of everyday life [reportedly related to immigrating to Canada]*”.

#### Time of day

The time of day for smoking was often mentioned by participants. They mentioned that the ‘morning cigarette’ was the most difficult one to quit. An English-speaking female participant stated that once she had the morning cigarette, she would begin to experience increased levels of craving throughout her day. Having a cigarette before going to bed was also mentioned. One male Chinese-speaking participant stated: “*The morning and before bed cigarettes are very important to me as to start and to end a day*”.

### Theme 4: Barriers to cessation

A majority of the participants did not know of the most effective cessation method(s) available to them and recommended for people in their age group, nor did they have many recommendations. However, the most common barriers to cessation included (a) lack of support; (b) lack of stress management skills; (c) traditional Chinese social norms; and (d) lack of motivation to quit.

#### Lack of support

Lack of support from family and friends was noted as a barrier to cessation. This was cited by smokers from both the English- and Chinese-speaking communities. Some of the participants in our study did not have quality, supportive relationships with their family members (e.g., the family were non-smokers and disapproved of smoking): “*My relationship with my other family members is not very good. They don’t want to understand me although they will be happy if I stopped smoking. I don’t think they will give me the support that I need*”. Several of the participants also had family who were not living in the same country (mainly Chinese-speaking participants) and, therefore, lacked any form of proximal familial support.

#### Lack of stress management skills

Life circumstances including health issues and life stressors were stated as major barriers to cessation by our participants. Stress from work, family, and relationships were mentioned as barriers to successful cessation: “*I relapsed due to stress related to changing jobs and my relationship with boyfriend*”. Quitting smoking was not a priority for some of the participants due to their current life situations as indicated by an English-speaking female participant: “*This not a good time to quitting [sic]*”. Another male participant said: “*I was on my own since I was sixteen and hanging out around that crowd that drank and smoked all the time. I just did the same thing as they did and wound up with COPD* [Chronic Obstructive Pulmonary Disease] *and I’m still smoking*”.

#### Traditional Chinese social norms

With regard to friends smoking and smoking as a social activity that triggers smoking, having a social network of friends or family who smoked was the greatest barrier to smoking cessation. This barrier was mentioned more frequently by Chinese-speaking participants than English-speaking participants. A Chinese-speaking male participant felt that his relationships with his friends would be damaged if he were to tell them that he was no longer smoking: “*It will hurt our relationship if I do so [tell friends I’m not around when they smoke]*”. Cessation was difficult for Chinese-speaking participants when they went back to visit their home country, China. Many of the Chinese-speaking participants mentioned that they would smoke more when returning to China as they were surrounded by smokers: “*It’s hard to resist if constantly being offer cigarettes [sic]*”. Similarly, a Chinese-speaking male participant stated that being in Canada made it inconvenient for him to smoke (due to smoking restrictions) and felt that being in Canada would help him to become smoke-free.

#### Lack of motivation to quit

Some of the participants mentioned that they could not quit smoking because they felt no real need or ‘no rock-solid reason’ to quit. This lack of motivation often stemmed from them not feeling any harmful effects to the body and not being able to observe any negative physiological signs or changes. An English-speaking female participant stated: “*I’d quit if I felt like crap but I don’t feel the urgency*”. Another Chinese-speaking male participant compared his situation to his grandfather who was also a heavy smoker and did not experience any smoking-related health issues: “*I am taking the chance of having the same genetic condition and luck as my grandfather*”. A female English-speaking smoker said: “*Worried the damage is done, irreversible, so it is hard for me to quit*”, indicating that she found no benefit in deciding to quit or cut down.

## Discussion

There is a need to provide smoking cessation consultation sessions to members of Chinese-Canadian communities, as Chinese-Canadians represent the largest group of ethnic populations in Canada and they have among the highest smoking prevalence of ethnocultural groups. Despite this evident need, few intervention studies have exclusively targeted Chinese-Canadians [20–22]. Our smoking cessation program was the first program developed to specifically meet the cultural and linguistic needs of Chinese Mandarin- and Cantonese-speaking communities.

In this feasibility study, we have learned several lessons that likely have implications for clinicians and researchers interested in reducing tobacco use among Chinese-Canadian communities. Our smoking cessation study was novel and innovative as we applied the following features: (1) culturally and linguistically sensitive consultations; (2) individualized treatment (action) plans; (3) a team-based approach including a review of each case between consultation sessions by members of the cessation team (e.g., counsellors, educators, nurses, researchers); (4) involvement of family members/next of kin in the smoking cessation process to gain their insights and support; (5) behavioural modification and risk perception concepts to improve participants’ self-confidence; (6) consultations in locations and at times more accessible to the participants (i.e. considering their needs and priorities); and (7) a variety of accessible communication methods (e.g., Short Message Service (SMS), e-mail, phone calls) to interact with participants for further support between consultations.

We recommend policy makers and stakeholders to be mindful when looking to implement various smoking cessation programs by considering potential cultural and linguistic barriers. Our study incorporated social and cultural factors—which were deemed critical to the cessation process for Chinese-speaking smokers by community members and key informants throughout our study’s development and consultation sessions. These factors included traditional Chinese values, the social acceptability of smoking, coping with immigration stress, the availability of social support, limited language proficiency to communicate in a new society, and the stigmatization of seeking smoking cessation services in their community (i.e., the factors that may pose additional barriers to smoking cessation). In order to provide services that can further respond to this nature of the requirement, an e-health platform with real time communication could possibly bridge the gap of this need [29–31].

The community-based participatory research (CBPR) approach utilized in our study—from the development of the study assessments and study protocol to the application of the counselling sessions—ensured that our smoking cessation program was best suited to the needs of participants from the target communities. This approach is fundamentally important in ensuring the target community is involved in important decisions and informing researchers and practitioners of components of their health that might not be readily apparent to clinicians and researchers in a traditional clinical setting (e.g., doctors office). Participants reported that our culturally and linguistically sensitive consultations made them feel more engaged than consultations they had participated in which did not utilize the CBPR approach. They reported feeling that they were an active part of the program, and respected. Moreover, the welcoming, relaxed atmosphere that we endeavored to create in all consultations and assessments likely deepened the level of conversation and may have made the participants more comfortable communicating their individual, and potentially sensitive, needs. Likewise, it also broadened the participants’ perspective with regards to finding the best ways to quit smoking because they felt more confident and, therefore, communicated effectively with the smoking cessation counsellor from their own culture.

The culturally related behaviours and patterns of smoking among Chinese-speaking participants emerged during data analysis. An obvious situation regarding the culture of smoking in China was that it is a common practice to offer and share cigarettes among individuals as a showing of respect and as a socialization tool [32–34]. This phenomenon does not exist to nearly the same extent in Western culture and may be difficult to describe and understand for individuals from different cultures or language groups. In our study, we acknowledged Chinese social cultures and customs, and our counsellors (from the same ethnic background as participants) were sensitive to these factors during all discussions. Taking the Chinese tradition of cigarette sharing into consideration, the counsellors worked with participants to develop alternative methods and action plans to turn down cigarette offerings, in a culturally sensitive manner. However, it is important to note that a cultural norm is difficult to change in a short period of time, and also varies between individuals within the same culture. In addition, we found that the social pressures of smoking, as a cultural norm, had greater impact on males than on females. Also, seeking assistance for quitting or reducing smoking is generally frowned upon in this culture. Both of these aspects may serve as barriers to participating in smoking cessation programs, especially those that mainly provide services in English. Accessibility to our culturally familiar smoking cessation programs (provided in their language by professionals from their community) led to noticeable changes in smoking perceptions and behaviours among Chinese-speaking participants in our study.

Another interesting finding was that quitting smoking did not appear to be a priority for some participants, though most individuals in the cohort identified smoking as being bad for their health. We assumed that participants of this study showing initiative to quit smoking would want to actively take measures to quit; however, as most of the Chinese-speaking smokers in our study were new immigrants, they were often busy with work, school, and adjusting to a new environment. Therefore, it was difficult for some of the participants to allocate a time during the day to attend the smoking cessation program, and quitting smoking was not prioritized. To combat this issue, our team introduced flexible scheduling to allow consultations and follow-ups to occur at a time and place most convenient for participants. We also found that providing responses to questions and support that smokers were seeking, in a timely manner, was crucial at the early stages of cutting down or quitting smoking. This may be especially important for smokers who are living alone and do not have the proximal social support that they need [35, 36].

### Implications

The feedback and data collected during the assessments from both study groups, surfaced three major implications for improving the: (1) availability, (2) accessibility, and (3) practicality of future smoking cessation educational programs for Chinese-Canadians.

#### Availability

Our study developed a first-of-its-kind project (culturally and linguistically appropriate smoking cessation program) in the Greater Vancouver Area and assisted Chinese-Canadians who were interested in quitting smoking. Family members and next of kin were also invited to attend consultation sessions and to share their thoughts and ideas all while providing their support.

#### Accessibility

We aimed to make our smoking cessation program accessible to as many smokers from the target communities who were willing to quit smoking as possible. As a result, we made an effort to meet participants at a time and place convenient for them and provided various accessible communication methods to contact the counsellors (e.g., digital media (e-mail, SMS, phone) or in-person).

#### Practicality

Our smoking cessation program provided information developed in the participants’ language and with consideration for culture norms. Our consultations were provided by healthcare professionals from their cultural group in a private and confidential environment. Moreover, individualized quit smoking action plans were developed with direct involvement of the participants, enhancing self-efficacy. Future studies can and should develop culturally and linguistically appropriate smoking cessation intervention programs for ethnocultural communities.

## Limitations

The major limitation of this study was related to recruitment of female smokers from the Chinese communities. In our sample, we recruited two female Chinese-speaking participants, which could affect the study results since the recommended intervention may not have similar findings with Chinese-speaking females. Moreover, having only two female Chinese-speaking participants does not provide rich enough data to identify themes for these participants; however, due to the smoking demographics from this community (e.g., the majority of Chinese Canadians smokers are men), and prevailing negative cultural attitudes toward females smoking in Chinese community, it is perhaps unsurprising that the majority of Chinese-speaking smokers recruited to participate in our study were males. Nonetheless, we believe that better recruitment approaches could be applied to enroll more female participants from Chinese communities (e.g., outreach and recruitment from the same gender or community) for future studies. In addition, the recruitment was conducted through convenience sampling, and is therefore not representative or generalizable to the Chinese smoking community at large. Another potential limitation is the lack of random assignment in our study. Since random assignment was not feasible, participants were asked to self-allocate themselves to either the intervention or the control group. Although smoking cessation outcomes were not the focus of this qualitative manuscript, a lack of random assignment might be a source of bias in our quantitative findings. Moreover, as this study was reliant on participant self-reported data, there is inherent subjectivity and social desirability biases associated with the responses given at each of the assessments. Lastly, another limitation is the application of thematic analysis and constant comparison for data analysis as it is subjective and relies on researcher’s judgement, potentially enabling researcher bias.

## Conclusion

From our previous research on ethnocultural communities, we have learned that a large part of the Chinese-Canadian population in the Greater Vancouver Area, similar to other new immigrant groups in Canada, are less likely to report having considered a physician’s advice to quit smoking than those who have not recently immigrated. In our study, Chinese-speaking participants were generally less likely than English-speaking participants to report that they would attempt to quit smoking based on the advice and education received from the media and health professionals, especially information regarding the link between smoking and cancer or heart and lung diseases. In terms of the effectiveness of the smoking cessation counselling program on participants’ risk perception and attitudes toward smoking, the participants maintained high levels of self-efficacy (e.g., understanding the importance of smoking cessation and confidence to quit smoking) at both 6-month and 8-month assessments. We also noticed that our smoking cessation program was generally accepted by participants in both language groups and participants reported that they were able to apply strategies learned in the consultations during their quit smoking journey. The fact that nearly all study participants had shown some movement towards reduction in the rate of smoking or complete cessation may indicate that our program was perceived as trustworthy and was thus accepted by the smokers who participated in the study. Given the importance of risk perceptions to change smoking behaviours and the unique characteristics of new Chinese immigrants in Canada, our study provided knowledge and information to examine the role of risk perceptions in smoking cessation among Chinese-speaking smokers. Such information can be applied in the development of further interventions that increase perceived susceptibility (realization of the possible harms of smoking) in order to promote smoking cessation among Chinese and other ethnic minority communities within British Columbia and across Canada. We consider this a feasibility study and the results will be applied as a basis for a large-scale intervention with combined culturally and linguistically sensitive smoking cessation and educational components for Chinese and other ethnocultural groups.

## Data Availability

The data collected and analyzed for the current study are available from the senior author (Iraj) upon reasonable request.

## Abbreviations

NRT: Nicotine Replacement Therapy
CBPR: community-based participatory research
NVivo: Navigating Viewpoints, Images and Value Observed
COPD: Chronic Obstructive Pulmonary Disease
SMS: Short Message Service
UBC: University of British Columbia

## References

[1] Fast Facts | Fact Sheets | Smoking & Tobacco Use | CDC. Centers for Disease Control and Prevention. Centers for Disease Control and Prevention; 2019. https://www.cdc.gov/tobacco/data_statistics/fact_sheets/fast_facts/index.htm. Accessed 1 Aug 2019.

[2] Smoking and Mortality. Health Canada 2008 Report. Canada.ca. Government of Canada; 2011. https://www.canada.ca/en/health-canada/services/health-concerns/tobacco/legislation/tobacco-product-labelling/smoking-mortality.html. Accessed 1 Aug 2019.

[3] Dobrescu A, Bhandari A, Sutherland G, Dinh T. The Costs of Tobacco Use in Canada, 2012. The Costs of Tobacco Use in Canada, 2012 - Canada.ca. Government of Canada; 2017. https://www.canada.ca/en/health-canada/services/publications/healthy-living/costs-tobacco-use-canada-2012.html. Accessed 1 Aug 2019.

[4] Tobacco Fact Sheets. World Health Organization. World Health Organization; 2019. https://www.who.int/news-room/fact-sheets/detail/tobacco. Accessed 1 Aug 2019.

[5] Neligan D. Smoking prevalence and cessation amongst immigrants to Canada: Canadian Research Data Centre Network. Smoking prevalence and cessation amongst immigrants to Canada. Canadian Research Data Centre Network; 2008. https://crdcn.org/smoking-prevalence-and-cessation-amongst-immigrants-canada. Accessed 1 Aug 2019.

[6] Immigration and Ethnocultural Diversity in Canada. Statistics Canada: Canada’s national statistical agency / Statistique Canada: Organisme statistique national du Canada. Statistics Canada; 2018. https://www12.statcan.gc.ca/nhs-enm/2011/as-sa/99-010-x/99-010-x2011001-eng.cfm. Accessed 1 Aug 2019.

[7] Mao A, Bottorff JL, Oliffe JL, Sarbit G, Kelly MT (2015). A qualitative study of Chinese Canadian fathers’ smoking behaviors: intersecting cultures and masculinities. BMC Public Health..

[8] Mao A, Bottorff JL (2016). A Qualitative study on unassisted smoking cessation among Chinese Canadian Immigrants. Am J Mens Health.

[9] Mao A, Bottorff JL, Oliffe JL, Sarbit G, Kelly MT (2016). A qualitative study on Chinese Canadian male immigrants’ perspectives on stopping smoking: implications for tobacco control in China. Am J Mens Health.

[10] Saw A, Paterniti D, Fung L-C, Tsoh JY, Chen MS, Tong EK (2016). Social environmental influences on smoking and cessation: qualitative perspectives among chinese-speaking smokers and non-smokers in California. J Immigrant Minority Health.

[11] Wu D, Ma GX, Zhou K, Zhou D, Liu A, Poon AN (2009). The effect of a culturally tailored smoking cessation for Chinese American smokers. Nicotine Tobacco Res..

[12] Abdullah ASM, Ho WWN (2006). What Chinese adolescents think about quitting smoking: a qualitative study. Substance Use Misuse..

[13] Bai X, Chen J-Y, Fang Z, Zhang X-Y, Wang F, Pan Z-Q (2017). Motivations, challenges and coping strategies for smoking cessation: based on multi-ethnic pregnant couples in far western China. J Huazhong Univ Sci Technol..

[14] Cai L-B, Xu F-R, Cheng Q-Z, Zhan J, Xie T, Ye Y-L (2015). Social smoking and mental health among Chinese male college students. Am J Health Prom.

[15] Chen Z, Peto R, Zhou M, Iona A (2015). Contrasting male and female trends in tobacco-attributed mortality in China: evidence from successive nationwide prospective cohort studies. Lancet..

[16] Koplan J, Eriksen M (2015). Smoking cessation for Chinese men and prevention for women. Lancet..

[17] Liu S, Zhang M, Yang L (2016). Prevalence and patterns of tobacco smoking among Chinese adult men and women: findings of the 2010 national smoking survey. J Epidemiol Community Health..

[18] Pollock G, Newbold BK, Lafrenière G, Edge S. Discrimination in the Doctor’s Office: Immigrants and Refugee Experiences | Critical Social Work - University of Windsor. University of Windsor; 2012. http://www1.uwindsor.ca/criticalsocialwork/discriminationindoctoroffice.

[19] Whittal A, Lippke S (2016). Investigating patients with an immigration background in Canada: relationships between individual immigrant attitudes, the doctor-patient relationship, and health outcomes. BMC Public Health..

[20] Poureslami IM, Shum J, Cheng N, Fitzgerald JM (2014). Does culture or illness change a smokers perspective on cessation?. Am J Health Behav..

[21] Fitzgerald JM, Poureslami I, Shum J. Assessing beliefs and risk perceptions on smoking and smoking cessation in immigrant Chinese adult smokers residing in Vancouver, Canada: a cross-sectional study. BMJ Open. 2015;5(2). https://bmjopen.bmj.com/content/5/2/e006435. 10.1136/bmjopen-2014-006435 PMC4322197 25649211

[22] Poureslami I, Shum J, Fitzgerald JM (2015). Why do Chinese people with COPD continue smoking: the attitudes and beliefs of Chinese residents of Vancouver, Canada?. Diversity Equal Health Care..

[23] Ranney L, Melvin C, Lux L, Mcclain E, Lohr KN. Systematic review: smoking cessation intervention strategies for adults and adults in special populations. Ann Intern Med. 2006;145(11):845. https://annals.org/aim/fullarticle/730874/systematic-review-smoking-cessation-intervention-strategies-adults-adults-special-populations. 10.7326/0003-4819-145-11-200612050-00142 16954352

[24] Hagens P, Pieterse M, Valk PVD, Palen JVD (2017). Effectiveness of intensive smoking reduction counselling plus combination nicotine replacement therapy in promoting long-term abstinence in patients with chronic obstructive pulmonary disease not ready to quit smoking: protocol of the REDUQ trial. Contemp Clin Trials Commun..

[25] Jiang B, He Y, Zuo F, Wu L, Liu Q-H, Zhang L, et al. Effectiveness of varenicline and counselling for smoking cessation in an observational cohort study in China. BMJ Open. 2016;6(1). https://bmjopen.bmj.com/content/6/1/e009381. 10.1136/bmjopen-2015-009381 PMC4716213 26739730

[26] Lancaster T, Stead LF. Individual behavioural counselling for smoking cessation. Cochrane Database Syst Rev. 2017. https://www.cochranelibrary.com/cdsr/doi/10.1002/14651858.CD001292.pub3/epdf/full. 10.1002/14651858.CD001292 12137623

[27] Cohen B, Schultz A, Walsh R. Exploring issues of equity within Canadian tobacco control initiatives: an environmental scan. University of Manitoba Faculty of Nursing. University of Manitoba; 2011. https://umanitoba.ca/faculties/nursing/media/issues_of_equity.pdf. Accessed 1 Aug 2019

[28] NVivo qualitative data analysis software; QSR International Pty Ltd. Version 12, 2018. https://www.qsrinternational.com/nvivo/home.

[29] Vidrine JI, Shete S, Cao Y, Greisinger A, Harmonson P, Sharp B, et al. Ask-Advise-Connect. JAMA Internal Med. 2013;173(6):458. https://jamanetwork.com/journals/jamainternalmedicine/fullarticle/1656544. 10.1001/jamainternmed.2013.3751 PMC3858085 23440173

[30] Wu L, He Y, Jiang B, Zuo F, Liu Q, Zhang L (2016). Effectiveness of additional follow-up telephone counseling in a smoking cessation clinic in Beijing and predictors of quitting among Chinese male smokers. BMC Public Health..

[31] Wu L, He Y, Jiang B, Zhang D, Tian H, Zuo F (2017). Very brief physician advice and supplemental proactive telephone calls to promote smoking reduction and cessation in Chinese male smokers with no intention to quit: a randomized trial. Addiction..

[32] Li J, Collins D (2015). Smoking environments in transition: the experiences of recent Chinese migrants to Canada. Health Soc Care Commun.

[33] Im PK, Mcneill A, Thompson ME, Fong GT, Xu S, Quah ACK, et al. Individual and interpersonal triggers to quit smoking in China: a cross-sectional analysis. Tobacco Control. 2015;24(Supplement 4): iv40–iv47. https://tobaccocontrol.bmj.com/content/24/Suppl_4/iv40. 10.1136/tobaccocontrol-2014-052198 PMC4644698 25888422

[34] Liu S, Zhang M, Yang L, Li Y, Wang L, Huang Z, et al. Prevalence and patterns of tobacco smoking among Chinese adult men and women: findings of the 2010 national smoking survey. J Epidemiol Community Health. 2017;71(2):154–61. https://jech.bmj.com/content/71/2/154. 10.1136/jech-2016-207805 PMC5284482 27660401

[35] Yun EH, Kang YH, Lim MK, Oh J-K, Son JM (2010). The role of social support and social networks in smoking behavior among middle and older aged people in rural areas of South Korea: a cross-sectional study. BMC Public Health..

[36] Westmaas JL, Bontemps-Jones J, Bauer JE. Social support in smoking cessation: reconciling theory and evidence. Nicotine Tobacco Res. 2010;12(7):695–707. https://academic.oup.com/ntr/article/12/7/695/1322455. 10.1093/ntr/ntq077 20513695

